# Crystal structure of {(*E*)-4-[(1-allyl-1*H*-1,2,3-triazol-4-yl)meth­oxy]benzyl­idene}[2-(morpholin-4-yl)eth­yl]amine

**DOI:** 10.1107/S1600536814016754

**Published:** 2014-08-01

**Authors:** Sevim Türktekin Çelikesir, Mehmet Akkurt, Aliasghar Jarrahpour, Mehdi Mohammadi Chermahini, Pezhman Shiri, Orhan Büyükgüngör

**Affiliations:** a Department of Physics, Faculty of Sciences, Erciyes University, 38039 Kayseri, Turkey; b Department of Chemistry, College of Sciences, Shiraz University, 71454 Shiraz, Iran; c Department of Physics, Faculty of Arts and Sciences, Ondokuz Mayıs University, 55139 Samsun, Turkey

**Keywords:** crystal structure, Schiff base, morpholine, 1,2,3-triazole, disorder

## Abstract

In the title compound, C_19_H_25_N_5_O_2_, the morpholine ring has a chair conformation. The plane of the central benzene ring makes dihedral angles of 88.75 (12) and 60.02 (7)°, respectively, with the mean plane formed by the four planar C atoms of the morpholine ring and with the plane of the triazole ring. In the crystal, mol­ecules are linked *via* C—H⋯π inter­actions, forming slabs lying parallel to (10-1). The C atoms of the bridging ethyl­ene group, between the morpholine and benzene rings, and the terminal ethene group of the prop-1-ene substituent attached to the triazole ring, are disordered over two sets of sites, with an occupancy ratio of 0.634 (13):0.366 (13).

## Related literature   

For information on Schiff bases, see: Vladimirova *et al.* (2001 ▶). For 1,2,3-triazole derivatives and the ‘click’ chemistry concept, see: Kolb *et al.* (2001 ▶); Wang *et al.* (2005 ▶). For the biological activity of 1,2,3-triazole derivatives, including their potential applications as anti­tumor, anti­bacterial, anti­fungal and anti­viral agents, see: Yu *et al.* (2006 ▶). For the biological utility of mol­ecules containing the morpholine moiety, see: Nelson *et al.* (2004 ▶).
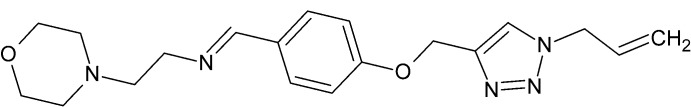


## Experimental   

### Crystal data  

C_19_H_25_N_5_O_2_
*M* *_r_* = 355.44
Monoclinic, 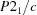

*a* = 22.3034 (10) Å
*b* = 5.2531 (3) Å
*c* = 16.5878 (8) Å
β = 93.051 (4)°
*V* = 1940.71 (17) Å^3^
*Z* = 4
Mo *K*α radiation
μ = 0.08 mm^−1^
*T* = 296 K
0.61 × 0.43 × 0.10 mm

### Data collection  

Stoe IPDS 2 diffractometer
Absorption correction: integration (*X-RED32*; Stoe & Cie, 2002 ▶) *T*_min_ = 0.961, *T*_max_ = 0.993
25155 measured reflections
3610 independent reflections
1769 reflections with *I* > 2σ(*I*)
*R*_int_ = 0.191

### Refinement  

*R*[*F* ^2^ > 2σ(*F* ^2^)] = 0.054
*wR*(*F* ^2^) = 0.130
*S* = 0.93
3610 reflections
272 parameters
7 restraints
H-atom parameters constrained
Δρ_max_ = 0.16 e Å^−3^
Δρ_min_ = −0.14 e Å^−3^

### 

Data collection: *X-AREA* (Stoe & Cie, 2002 ▶); cell refinement: *X-AREA*; data reduction: *X-RED32* (Stoe & Cie, 2002 ▶); program(s) used to solve structure: *SHELXS97* (Sheldrick, 2008 ▶); program(s) used to refine structure: *SHELXL97* (Sheldrick, 2008 ▶); molecular graphics: *ORTEP-3 for Windows* (Farrugia, 2012 ▶); software used to prepare material for publication: *WinGX* (Farrugia, 2012 ▶) and *PLATON* (Spek, 2009 ▶).

## Supplementary Material

Crystal structure: contains datablock(s) global, I. DOI: 10.1107/S1600536814016754/su2753sup1.cif

Structure factors: contains datablock(s) I. DOI: 10.1107/S1600536814016754/su2753Isup2.hkl

Supporting information file. DOI: 10.1107/S1600536814016754/su2753Isup3.cml

**Fig. 1.**
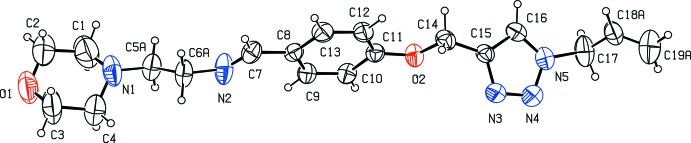
The molecular structure of the title molecule, with atom labelling. The displacement ellipsoids are drawn at the 30% probability level. For clarity only the major disordered components are shown.

**Fig. 2.**
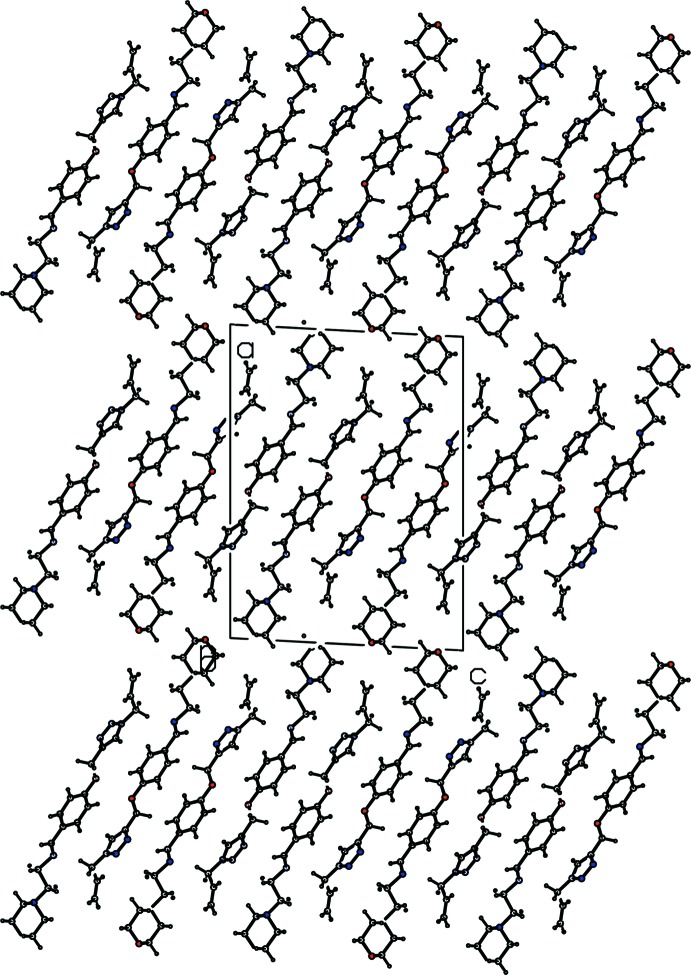
Crystal packing diagram of the title compound viewed along the b axis. For clarity only the major disordered components are shown.

. DOI: 10.1107/S1600536814016754/su2753fig1.tif
The mol­ecular structure of the title mol­ecule, with atom labelling. The displacement ellipsoids are drawn at the 30% probability level. For clarity only the major disordered components are shown.

b . DOI: 10.1107/S1600536814016754/su2753fig2.tif
Crystal packing diagram of the title compound viewed along the *b* axis. For clarity only the major disordered components are shown.

CCDC reference: 1014976

Additional supporting information:  crystallographic information; 3D view; checkCIF report

## Figures and Tables

**Table 1:** Hydrogen-bond geometry (Å, °) *Cg* is the centroid of the 1,2,3-triazole ring N3–N5/C15/C16.

*D*—H⋯*A*	*D*—H	H⋯*A*	*D*⋯*A*	*D*—H⋯*A*
C6*A*—H6*A*1⋯*Cg* ^i^	0.97	2.90	3.617 (8)	132

Symmetry code: (i) 
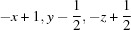
.

## supplementary crystallographic information

### S1. Synthesis and crystallization

Reaction of 4-((1-allyl-1H-1,2,3-triazol-4-yl)meth­oxy)­benzaldehyde (1.0 mmol) with 2-morpholino­ethanamine (1.0 mmol) in refluxing ethanol gave the title compound. Recrystallization from ethanol gave light brown crystals in 75% yield. m.p. 385 - 387 K. IR (KBr, cm^-1^): 1635 (C=N). ^1^H-NMR (250 MHz, CDCl_3_), δ (ppm): 2.48 (CH_2_—N morpholine, t, 4H, J=5 Hz), 2.62 (morpholine-CH_2_—CH_2_, t, 2H, J=7.5 Hz), 3.65 (CH_2_—O morpholine and morpholine-CH_2_—CH_2_, t, 6H, J=5 Hz), 4.92 (d, 2H, J=7.5 Hz), 5.17 (s, 2H), 5.29 (m, 2H), 5.98 (m, 1H), 6.92 (aromatic H, d, 2H, J=10 Hz), 7.56 (aromatic H, d, 2H, J= Hz 5), 7.6 (H triazole, s, 1H), 8.15 (HC═N, s, 1H). ^13^CNMR (62.9 MHz, CDCl_3_), δ (p.p.m): 53.9 (CH_2_—N morpholine), 58.8, 59.3 (N—CH_2_—CH_2_, and CH_2_—N), 61.9 (CH_2_—O), 66.9 (CH_2_—O morpholine), 114.7-143.9 (aromatic carbons and C=C triazole), 161.1 (C=N).

### S2. Refinement

Crystal data, data collection and structure refinement details are summarized in Table 2. All H atoms were included in calculated positions and treated as riding atoms: C—H = 0.93 - 0.97 Å with *U*_iso_(H) = 1.2*U*_eq_(C). Reflections (1 2 0), (1 3 0) and (0 4 1) were omitted owing to bad agreement. Atoms C5A/C5B, C6A/C6B, C18A/C18B and C19A/C19B are disordered over two sites with an occupancy ratio of 0.634 (13):0.366 (13). Owing to the poor quality of the crystal the value of *R*_int_ is high (0.191).

### Crystal data

**Table:**

C_19_H_25_N_5_O_2_	*F*(000) = 760
*M**_r_* = 355.44	*D*_x_ = 1.217 Mg m^−^^3^
Monoclinic, *P*2_1_/*c*	Mo *K*α radiation, λ = 0.71073 Å
Hall symbol: -P 2ybc	Cell parameters from 15061 reflections
*a* = 22.3034 (10) Å	θ = 1.5–27.1°
*b* = 5.2531 (3) Å	µ = 0.08 mm^−^^1^
*c* = 16.5878 (8) Å	*T* = 296 K
β = 93.051 (4)°	Block, light brown
*V* = 1940.71 (17) Å^3^	0.61 × 0.43 × 0.10 mm
*Z* = 4	

### Data collection

**Table:**

Stoe IPDS 2 diffractometer	3610 independent reflections
Radiation source: sealed X-ray tube, 12 x 0.4 mm long-fine focus	1769 reflections with *I* > 2σ(*I*)
Plane graphite monochromator	*R*_int_ = 0.191
Detector resolution: 6.67 pixels mm^-1^	θ_max_ = 25.5°, θ_min_ = 1.8°
ω scans	*h* = −26→26
Absorption correction: integration (*X-RED32*; Stoe & Cie, 2002)	*k* = −6→6
*T*_min_ = 0.961, *T*_max_ = 0.993	*l* = −20→20
25155 measured reflections	

### Refinement

**Table:**

Refinement on *F*^2^	7 restraints
Least-squares matrix: full	Hydrogen site location: inferred from neighbouring sites
*R*[*F*^2^ > 2σ(*F*^2^)] = 0.054	H-atom parameters constrained
*wR*(*F*^2^) = 0.130	*w* = 1/[σ^2^(*F*_o_^2^) + (0.0624*P*)^2^] where *P* = (*F*_o_^2^ + 2*F*_c_^2^)/3
*S* = 0.93	(Δ/σ)_max_ = 0.001
3610 reflections	Δρ_max_ = 0.16 e Å^−^^3^
272 parameters	Δρ_min_ = −0.14 e Å^−^^3^

### Special details

**Table:**

Geometry. Bond distances, angles *etc*. have been calculated using the rounded fractional coordinates. All su's are estimated from the variances of the (full) variance-covariance matrix. The cell e.s.d.'s are taken into account in the estimation of distances, angles and torsion angles
Refinement. Refinement on *F*^2^ for ALL reflections except those flagged by the user for potential systematic errors. Weighted *R*-factors *wR* and all goodnesses of fit *S* are based on *F*^2^, conventional *R*-factors *R* are based on *F*, with *F* set to zero for negative *F*^2^. The observed criterion of *F*^2^ > σ(*F*^2^) is used only for calculating -*R*-factor-obs *etc*. and is not relevant to the choice of reflections for refinement. *R*-factors based on *F*^2^ are statistically about twice as large as those based on *F*, and *R*-factors based on ALL data will be even larger.

### Fractional atomic coordinates and isotropic or equivalent isotropic displacement parameters (Å^2^)

**Table:**

	*x*	*y*	*z*	*U*_iso_*/*U*_eq_	Occ. (<1)
O1	0.00977 (10)	0.1772 (6)	0.1080 (2)	0.1613 (15)	
O2	0.52892 (6)	0.7614 (3)	0.42272 (9)	0.0777 (6)	
N1	0.12323 (13)	0.3855 (6)	0.15681 (19)	0.1249 (11)	
N2	0.27681 (10)	0.5151 (6)	0.24066 (15)	0.1151 (10)	
N3	0.65963 (9)	0.6517 (4)	0.44128 (13)	0.0839 (8)	
N4	0.70715 (9)	0.6485 (4)	0.49113 (14)	0.0923 (9)	
N5	0.70797 (8)	0.8676 (4)	0.53186 (12)	0.0807 (8)	
C1	0.0707 (2)	0.4126 (9)	0.2028 (3)	0.172 (2)	
C2	0.01503 (16)	0.4008 (10)	0.1505 (3)	0.179 (3)	
C3	0.05926 (16)	0.1478 (10)	0.0616 (3)	0.180 (2)	
C4	0.11659 (13)	0.1510 (8)	0.1120 (2)	0.1436 (18)	
C5A	0.1714 (3)	0.4194 (16)	0.2175 (5)	0.096 (2)	0.634 (13)
C6A	0.2293 (3)	0.482 (2)	0.1740 (5)	0.096 (3)	0.634 (13)
C7	0.31518 (10)	0.6820 (5)	0.23080 (15)	0.0809 (10)	
C8	0.37040 (9)	0.7061 (4)	0.28150 (13)	0.0658 (8)	
C9	0.38592 (9)	0.5339 (4)	0.34293 (14)	0.0711 (8)	
C10	0.43840 (9)	0.5580 (4)	0.38852 (14)	0.0695 (8)	
C11	0.47744 (9)	0.7556 (4)	0.37429 (13)	0.0646 (8)	
C12	0.46337 (10)	0.9286 (4)	0.31340 (14)	0.0774 (9)	
C13	0.40997 (11)	0.9006 (4)	0.26846 (15)	0.0805 (9)	
C14	0.57484 (10)	0.9369 (4)	0.40298 (15)	0.0775 (9)	
C15	0.63014 (9)	0.8731 (4)	0.45100 (13)	0.0644 (8)	
C16	0.66101 (11)	1.0086 (5)	0.50812 (15)	0.0795 (9)	
C17	0.75478 (13)	0.9218 (7)	0.59454 (18)	0.1190 (14)	
C18A	0.7974 (4)	1.113 (2)	0.5714 (7)	0.114 (3)	0.634 (13)
C19A	0.8541 (6)	1.080 (3)	0.5825 (11)	0.151 (5)	0.634 (13)
C5B	0.1961 (5)	0.338 (2)	0.1835 (7)	0.083 (4)	0.366 (13)
C6B	0.2144 (7)	0.590 (3)	0.2047 (9)	0.104 (5)	0.366 (13)
C18B	0.8105 (6)	0.998 (4)	0.5629 (12)	0.109 (6)	0.366 (13)
C19B	0.8417 (14)	1.199 (3)	0.579 (2)	0.153 (10)	0.366 (13)
H1A	0.07240	0.57420	0.23110	0.2060*	
H1B	0.07020	0.27810	0.24280	0.2060*	
H5A1	0.17700	0.26510	0.24920	0.1160*	0.634 (13)
H3A	0.05980	0.28410	0.02220	0.2160*	
H3B	0.05590	−0.01220	0.03260	0.2160*	
H6A2	0.22430	0.63690	0.14260	0.1150*	0.634 (13)
H5A2	0.16200	0.55730	0.25360	0.1160*	0.634 (13)
H2A	−0.01930	0.41900	0.18370	0.2150*	
H2B	0.01450	0.54230	0.11290	0.2150*	
H6A1	0.23960	0.34400	0.13840	0.1150*	0.634 (13)
H10	0.44790	0.44120	0.42930	0.0830*	
H12	0.48940	1.06130	0.30290	0.0930*	
H13	0.40040	1.01780	0.22780	0.0970*	
H14A	0.56250	1.10930	0.41490	0.0930*	
H14B	0.58190	0.92630	0.34590	0.0930*	
H16	0.65130	1.16920	0.52710	0.0950*	
H17A	0.73600	0.97870	0.64280	0.1430*	
H17B	0.77630	0.76560	0.60780	0.1430*	
H18A	0.78320	1.26370	0.54790	0.1370*	0.634 (13)
H19A	0.86880	0.93050	0.60600	0.1820*	0.634 (13)
H19B	0.88050	1.20590	0.56710	0.1820*	0.634 (13)
H4A	0.11720	0.00870	0.14930	0.1720*	
H4B	0.15010	0.13150	0.07760	0.1720*	
H7	0.30840	0.79780	0.18890	0.0970*	
H9	0.36010	0.40000	0.35310	0.0850*	
H5B1	0.20110	0.22290	0.22900	0.0990*	0.366 (13)
H5B2	0.21790	0.27290	0.13880	0.0990*	0.366 (13)
H6B1	0.18950	0.66640	0.24420	0.1250*	0.366 (13)
H6B2	0.21640	0.70040	0.15800	0.1250*	0.366 (13)
H18B	0.82620	0.88730	0.52550	0.1310*	0.366 (13)
H19C	0.82840	1.31630	0.61640	0.1840*	0.366 (13)
H19D	0.87750	1.22610	0.55460	0.1840*	0.366 (13)

### Atomic displacement parameters (Å^2^)

**Table:**

	*U*^11^	*U*^22^	*U*^33^	*U*^12^	*U*^13^	*U*^23^
O1	0.0905 (16)	0.195 (3)	0.196 (3)	−0.0408 (17)	−0.0134 (17)	−0.031 (2)
O2	0.0721 (9)	0.0850 (10)	0.0749 (11)	−0.0148 (8)	−0.0063 (8)	0.0196 (8)
N1	0.1009 (18)	0.141 (2)	0.127 (2)	−0.0382 (17)	−0.0480 (18)	0.0348 (19)
N2	0.0763 (14)	0.164 (2)	0.1018 (18)	−0.0213 (15)	−0.0260 (13)	0.0395 (17)
N3	0.0847 (13)	0.0753 (13)	0.0906 (16)	0.0021 (11)	−0.0044 (12)	−0.0121 (11)
N4	0.0861 (14)	0.0884 (15)	0.1015 (17)	0.0078 (12)	−0.0030 (13)	−0.0022 (13)
N5	0.0753 (13)	0.0914 (14)	0.0747 (14)	−0.0147 (12)	−0.0029 (11)	0.0018 (12)
C1	0.147 (3)	0.194 (4)	0.171 (4)	−0.033 (3)	−0.022 (3)	−0.030 (3)
C2	0.101 (3)	0.206 (5)	0.227 (5)	−0.006 (3)	−0.021 (3)	−0.035 (4)
C3	0.090 (2)	0.266 (5)	0.180 (4)	−0.035 (3)	−0.021 (3)	−0.065 (4)
C4	0.090 (2)	0.183 (4)	0.156 (3)	−0.008 (2)	−0.011 (2)	−0.001 (3)
C5A	0.066 (3)	0.141 (5)	0.082 (4)	−0.004 (3)	0.001 (3)	0.001 (3)
C6A	0.059 (3)	0.151 (7)	0.077 (5)	−0.011 (4)	0.000 (3)	−0.001 (4)
C7	0.0742 (15)	0.1012 (19)	0.0670 (16)	0.0125 (14)	0.0008 (13)	0.0074 (14)
C8	0.0673 (13)	0.0702 (14)	0.0597 (15)	0.0124 (11)	0.0007 (11)	−0.0038 (11)
C9	0.0664 (14)	0.0790 (14)	0.0680 (16)	−0.0028 (11)	0.0048 (12)	0.0050 (13)
C10	0.0686 (13)	0.0735 (14)	0.0664 (15)	−0.0002 (11)	0.0041 (12)	0.0155 (11)
C11	0.0681 (13)	0.0658 (13)	0.0594 (14)	0.0053 (11)	−0.0024 (11)	0.0016 (11)
C12	0.0843 (16)	0.0622 (14)	0.0844 (18)	−0.0044 (11)	−0.0085 (14)	0.0129 (12)
C13	0.0953 (17)	0.0662 (14)	0.0785 (18)	0.0044 (13)	−0.0104 (14)	0.0156 (12)
C14	0.0825 (15)	0.0649 (13)	0.0845 (18)	−0.0127 (11)	0.0003 (13)	0.0070 (12)
C15	0.0676 (13)	0.0558 (12)	0.0697 (15)	−0.0082 (11)	0.0032 (12)	0.0023 (11)
C16	0.0864 (16)	0.0665 (13)	0.0849 (18)	−0.0048 (13)	−0.0009 (14)	−0.0124 (13)
C17	0.0904 (19)	0.175 (3)	0.089 (2)	−0.029 (2)	−0.0193 (17)	0.002 (2)
C18A	0.094 (5)	0.092 (5)	0.152 (6)	−0.016 (5)	−0.042 (5)	−0.009 (5)
C19A	0.093 (6)	0.206 (13)	0.153 (7)	−0.026 (9)	−0.007 (5)	0.020 (11)
C5B	0.071 (7)	0.119 (7)	0.057 (6)	0.029 (5)	0.000 (5)	0.002 (5)
C6B	0.114 (10)	0.128 (10)	0.068 (8)	0.010 (8)	−0.008 (6)	0.006 (6)
C18B	0.066 (9)	0.125 (12)	0.134 (9)	0.009 (7)	−0.011 (6)	−0.027 (10)
C19B	0.116 (17)	0.127 (13)	0.217 (19)	−0.036 (11)	0.016 (14)	−0.018 (14)

### Geometric parameters (Å, º)

**Table:**

O1—C2	1.372 (6)	C1—H1A	0.9700
O1—C3	1.388 (5)	C1—H1B	0.9700
O2—C11	1.366 (2)	C2—H2A	0.9700
O2—C14	1.429 (3)	C2—H2B	0.9700
N1—C1	1.439 (5)	C3—H3A	0.9700
N1—C4	1.442 (5)	C3—H3B	0.9700
N1—C5A	1.444 (8)	C4—H4A	0.9700
N1—C5B	1.680 (12)	C4—H4B	0.9700
N2—C6A	1.500 (8)	C5A—H5A1	0.9700
N2—C7	1.242 (4)	C5A—H5A2	0.9700
N2—C6B	1.536 (16)	C5B—H5B1	0.9700
N3—N4	1.309 (3)	C5B—H5B2	0.9700
N3—C15	1.350 (3)	C6A—H6A2	0.9700
N4—N5	1.334 (3)	C6A—H6A1	0.9700
N5—C16	1.325 (3)	C6B—H6B2	0.9700
N5—C17	1.462 (4)	C6B—H6B1	0.9700
C1—C2	1.478 (6)	C7—H7	0.9300
C3—C4	1.490 (5)	C9—H9	0.9300
C5A—C6A	1.548 (10)	C10—H10	0.9300
C5B—C6B	1.424 (19)	C12—H12	0.9300
C7—C8	1.459 (3)	C13—H13	0.9300
C8—C9	1.392 (3)	C14—H14A	0.9700
C8—C13	1.375 (3)	C14—H14B	0.9700
C9—C10	1.365 (3)	C16—H16	0.9300
C10—C11	1.383 (3)	C17—H17A	0.9700
C11—C12	1.382 (3)	C17—H17B	0.9700
C12—C13	1.379 (3)	C18A—H18A	0.9300
C14—C15	1.471 (3)	C18B—H18B	0.9300
C15—C16	1.345 (3)	C19A—H19B	0.9300
C17—C18B	1.432 (15)	C19A—H19A	0.9300
C17—C18A	1.449 (10)	C19B—H19C	0.9300
C18A—C19A	1.280 (16)	C19B—H19D	0.9300
C18B—C19B	1.29 (3)		
			
C2—O1—C3	109.4 (3)	N1—C4—H4B	109.00
C11—O2—C14	118.02 (17)	C3—C4—H4A	109.00
C1—N1—C4	107.1 (3)	C3—C4—H4B	110.00
C1—N1—C5A	102.4 (4)	H4A—C4—H4B	108.00
C1—N1—C5B	132.7 (5)	N1—C5A—H5A1	110.00
C4—N1—C5A	121.1 (4)	N1—C5A—H5A2	110.00
C4—N1—C5B	94.6 (4)	C6A—C5A—H5A1	110.00
C6A—N2—C7	116.7 (4)	C6A—C5A—H5A2	110.00
C6B—N2—C7	112.6 (6)	H5A1—C5A—H5A2	108.00
N4—N3—C15	108.6 (2)	H5B1—C5B—H5B2	109.00
N3—N4—N5	107.31 (19)	N1—C5B—H5B2	112.00
N4—N5—C16	110.0 (2)	C6B—C5B—H5B1	112.00
N4—N5—C17	121.2 (2)	N1—C5B—H5B1	112.00
C16—N5—C17	128.7 (2)	C6B—C5B—H5B2	112.00
N1—C1—C2	111.5 (4)	N2—C6A—H6A2	111.00
O1—C2—C1	112.6 (4)	C5A—C6A—H6A1	111.00
O1—C3—C4	111.8 (4)	N2—C6A—H6A1	111.00
N1—C4—C3	111.1 (3)	C5A—C6A—H6A2	111.00
N1—C5A—C6A	108.0 (6)	H6A1—C6A—H6A2	109.00
N1—C5B—C6B	100.8 (9)	N2—C6B—H6B1	113.00
N2—C6A—C5A	104.8 (6)	C5B—C6B—H6B2	113.00
N2—C6B—C5B	95.7 (10)	N2—C6B—H6B2	113.00
N2—C7—C8	123.5 (2)	C5B—C6B—H6B1	113.00
C9—C8—C13	117.5 (2)	H6B1—C6B—H6B2	110.00
C7—C8—C9	122.3 (2)	N2—C7—H7	118.00
C7—C8—C13	120.2 (2)	C8—C7—H7	118.00
C8—C9—C10	121.17 (19)	C10—C9—H9	119.00
C9—C10—C11	120.3 (2)	C8—C9—H9	119.00
O2—C11—C12	124.47 (19)	C9—C10—H10	120.00
C10—C11—C12	119.8 (2)	C11—C10—H10	120.00
O2—C11—C10	115.68 (19)	C11—C12—H12	121.00
C11—C12—C13	118.7 (2)	C13—C12—H12	121.00
C8—C13—C12	122.5 (2)	C8—C13—H13	119.00
O2—C14—C15	108.63 (18)	C12—C13—H13	119.00
C14—C15—C16	130.1 (2)	O2—C14—H14A	110.00
N3—C15—C14	122.10 (19)	O2—C14—H14B	110.00
N3—C15—C16	107.8 (2)	C15—C14—H14A	110.00
N5—C16—C15	106.3 (2)	C15—C14—H14B	110.00
N5—C17—C18A	113.6 (5)	H14A—C14—H14B	108.00
N5—C17—C18B	113.3 (8)	C15—C16—H16	127.00
C17—C18A—C19A	121.7 (11)	N5—C16—H16	127.00
C17—C18B—C19B	129 (2)	N5—C17—H17A	109.00
N1—C1—H1A	109.00	C18A—C17—H17B	109.00
N1—C1—H1B	109.00	N5—C17—H17B	109.00
C2—C1—H1A	109.00	C18A—C17—H17A	109.00
C2—C1—H1B	109.00	C18B—C17—H17B	84.00
H1A—C1—H1B	108.00	H17A—C17—H17B	108.00
O1—C2—H2A	109.00	C18B—C17—H17A	130.00
O1—C2—H2B	109.00	C19A—C18A—H18A	119.00
C1—C2—H2A	109.00	C17—C18A—H18A	119.00
C1—C2—H2B	109.00	C19B—C18B—H18B	116.00
H2A—C2—H2B	108.00	C17—C18B—H18B	116.00
O1—C3—H3A	109.00	C18A—C19A—H19B	120.00
O1—C3—H3B	109.00	H19A—C19A—H19B	120.00
C4—C3—H3A	109.00	C18A—C19A—H19A	120.00
C4—C3—H3B	109.00	C18B—C19B—H19C	120.00
H3A—C3—H3B	108.00	C18B—C19B—H19D	120.00
N1—C4—H4A	109.00	H19C—C19B—H19D	120.00
			
C2—O1—C3—C4	57.7 (5)	N4—N5—C16—C15	0.0 (3)
C3—O1—C2—C1	−57.7 (5)	N1—C1—C2—O1	58.5 (5)
C14—O2—C11—C12	−8.4 (3)	O1—C3—C4—N1	−58.5 (5)
C11—O2—C14—C15	−167.78 (18)	N1—C5A—C6A—N2	179.8 (6)
C14—O2—C11—C10	170.68 (19)	N2—C7—C8—C13	−178.0 (3)
C1—N1—C4—C3	55.4 (4)	N2—C7—C8—C9	3.4 (4)
C4—N1—C5A—C6A	79.1 (7)	C7—C8—C13—C12	−178.5 (2)
C4—N1—C1—C2	−55.3 (5)	C7—C8—C9—C10	178.8 (2)
C5A—N1—C1—C2	176.3 (5)	C9—C8—C13—C12	0.1 (3)
C1—N1—C5A—C6A	−161.9 (6)	C13—C8—C9—C10	0.1 (3)
C5A—N1—C4—C3	172.1 (5)	C8—C9—C10—C11	−0.1 (3)
C6A—N2—C7—C8	−167.7 (4)	C9—C10—C11—C12	−0.3 (3)
C7—N2—C6A—C5A	−143.5 (5)	C9—C10—C11—O2	−179.44 (19)
C15—N3—N4—N5	−0.4 (3)	C10—C11—C12—C13	0.6 (3)
N4—N3—C15—C14	179.4 (2)	O2—C11—C12—C13	179.6 (2)
N4—N3—C15—C16	0.4 (3)	C11—C12—C13—C8	−0.5 (3)
N3—N4—N5—C16	0.3 (3)	O2—C14—C15—N3	65.1 (3)
N3—N4—N5—C17	178.3 (2)	O2—C14—C15—C16	−116.2 (3)
C17—N5—C16—C15	−177.8 (2)	N3—C15—C16—N5	−0.3 (3)
N4—N5—C17—C18A	108.4 (5)	C14—C15—C16—N5	−179.1 (2)
C16—N5—C17—C18A	−74.0 (5)	N5—C17—C18A—C19A	−132.7 (12)

### Hydrogen-bond geometry (Å, º)

Cg is the centroid of the 1,2,3-triazole ring N3–N5/C15/C16.

**Table:**

*D*—H···*A*	*D*—H	H···*A*	*D*···*A*	*D*—H···*A*
C6*A*—H6*A*1···*Cg*^i^	0.97	2.90	3.617 (8)	132

Symmetry code: (i) −*x*+1, *y*−1/2, −*z*+1/2.
