# Supplementary figures and images for: Crystal structure of {(E)-4-[(1-allyl-1H-1,2,3-triazol-4-yl)meth­oxy]benzyl­idene}[2-(morpholin-4-yl)eth­yl]amine
Source: Acta Crystallogr Sect E Struct Rep Online. 2014 Aug 1;70(Pt 9):o933–4. doi: 10.1107/S1600536814016754 (PMC4186139; doi:10.1107/S1600536814016754)

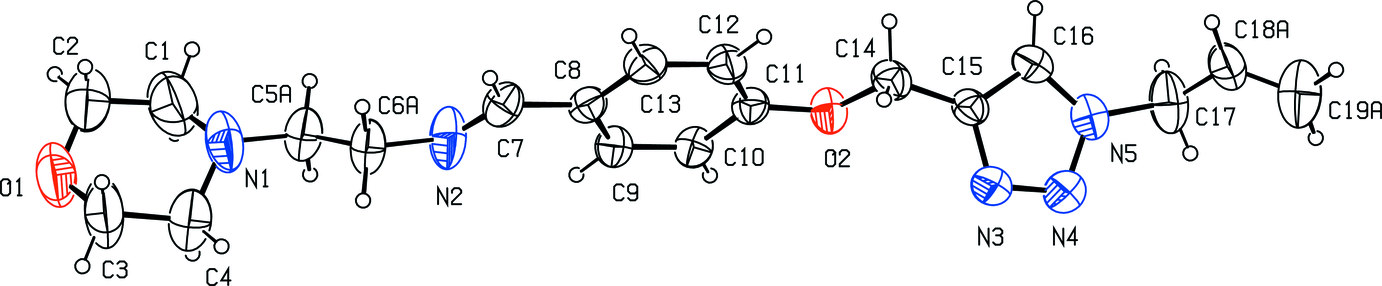

Supplement: Supplementary file 4 [file e-70-0o933-fig1.tif]

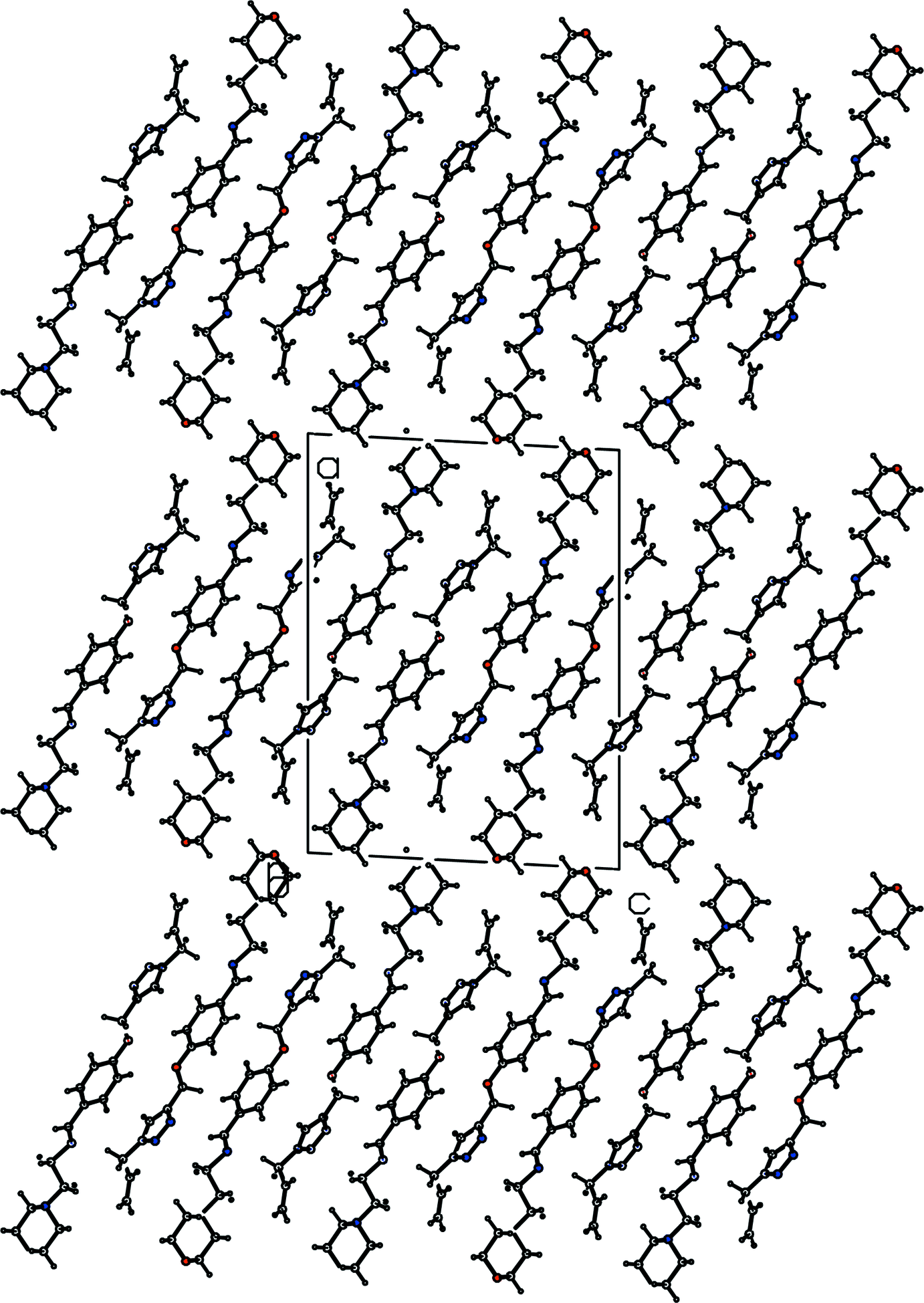

Supplement: Supplementary file 5 [file e-70-0o933-fig2.tif]
